# Penetration Depth and Tissue Interaction of Focused Extracorporeal Shock Waves: An In-vitro Investigation

**DOI:** 10.7759/cureus.80205

**Published:** 2025-03-07

**Authors:** Leandro R Iuamoto, Wu T Hsing

**Affiliations:** 1 Acupuncture Center, Instituto de Ortopedia e Traumatologia do Hospital das Clínicas da Faculdade de Medicina da Universidade de São Paulo, São Paulo, BRA

**Keywords:** bone and bones, extracorporeal shockwave therapy, high-energy shock waves, musculoskeletal system, superficial musculoaponeurotic system

## Abstract

Introduction: Extracorporeal shock wave therapy (ESWT) is a non-invasive therapeutic approach with minimal consequences, extensively utilized for the management of musculoskeletal problems. Focused ESWT (f-ESWT) is known to have regenerative effects on muscle and bone tissues; however, little is known about its penetration and propagation in different tissues.

Objective: To assess the reach of f-ESWT in soft tissues and bone tissues.

Methods: An in-vitro observational study was conducted. A piezoelectric shock wave device was used to evaluate the penetration and visual propagation of shock waves in an aquarium with water in muscle tissues (ham) of different thicknesses, and bone tissues (scapula and femur). High-resolution images and videos were captured to observe the penetration of mechanical waves into the different tissues.

Results: Light beams and air bubbles were observed, consistent with the propagation of f-ESWT through different tissues. The f-ESWT was able to penetrate wide bones such as the femur and thin bones such as the scapula.

Conclusion: F-ESWT is capable of penetrating soft tissues and bone tissues depending on the depth. This is an important study for the safe application of f-ESWT by a healthcare professional with prior anatomical knowledge.

## Introduction

Shock waves are physical phenomena characterized by propagating disturbances that cause variations in temperature, density, and pressure. They are generated when the velocity surpasses the speed of sound, resulting in a rapid increase in positive pressure (ranging from 5 to 120 MPa) within just 5 nanoseconds, followed by a subsequent drop to approximately -20 MPa [1,2].

The study of shock waves originated from aerospace research conducted between 1966 and 1972 when German engineers observed their effects on biological tissues. Initial experiments demonstrated their ability to fragment kidney stones in an open water bath, paving the way for the development of the first high-energy shock wave lithotripter (TM1), which was ultrasound-guided and introduced in 1974 [3-5].

By the 1980s, shock waves had become an established method for the disintegration of renal crystal aggregates, delivering promising clinical outcomes. From the 1990s onward, researchers shifted their focus to adapting this technology for the treatment of pathological neocalcifications associated with musculoskeletal disorders, such as calcific tendinopathy of the shoulder [6].

As a result, shock wave therapy was further developed for the management of chronic heel pain and later extended to plantar fasciitis, demonstrating superior efficacy compared to both conservative and surgical approaches. However, due to the potential risk of high-energy shock waves inducing tissue rupture, treatment intensities had to be adjusted to minimize the likelihood of tissue damage [2,7].

Extracorporeal shock wave therapy (ESWT) has emerged as a widely used non-invasive treatment modality for musculoskeletal disorders, offering a favorable safety profile with minimal complications [8-10]. This therapy can be delivered in two distinct modalities: radial (r-ESWT) and focused (f-ESWT) [11].

r-ESWT generates low-intensity shock waves with a divergent propagation pattern, where energy gradually decreases as it moves away from the point of origin. This mechanism induces transient pressure fluctuations, characterized by alternating increases and decreases in pressure. Unlike focused devices, radial systems produce pressure pulses rather than true shock waves, as they exhibit a slower rise time and lower peak pressure. Due to these properties, r-ESWT is primarily indicated for treating superficial soft tissue conditions, such as myotendinous injuries [12,13].

In contrast, f-ESWT utilizes mechanically generated acoustic waves that are concentrated at a specific focal zone, allowing for deeper tissue penetration. Studies have demonstrated that f-ESWT is capable of targeting structures such as bones, depending on the applied energy levels. The therapeutic efficacy of f-ESWT is largely determined by the energy flux density (EFD) at the focal point, measured in mJ/mm². Clinically, EFD values typically range from 0.001 to 0.5 mJ/mm² [13-15].

Focused shock waves propagate rapidly through space and have the potential to induce various biological responses. These waves play a key role in local biological modulation, promoting mesenchymal stem cell differentiation, neovascularization, and the release of angiogenic factors. Although the clinical success of f-ESWT is well documented, the underlying mechanisms remain an active area of research. f-ESWT facilitates energy transmission to cells, triggering effects such as increased cell membrane permeability, ionization of biological molecules, and activation of pain-modulating signal transduction pathways. These include mechanotransduction signaling, extracellular signal-regulated kinase (ERK) signaling, focal adhesion kinase (FAK) signaling, and toll-like receptor 3 (TLR3) signaling, all of which contribute to gene expression regulation [16,17].

Additionally, f-ESWT stimulates the release of adenosine triphosphate (ATP), which activates cellular signaling pathways. Shock waves influence ion channel functionality in the cell membrane, modulating calcium influx into cells. As a result, f-ESWT has been shown to regulate angiogenesis through factors such as von Willebrand factor (vWF), vascular endothelial growth factor (VEGF), endothelial nitric oxide synthase (eNOS), and proliferating cell nuclear antigen (PCNA), while also exerting anti-inflammatory effects by modulating soluble intercellular adhesion molecule-1 (sICAM) and soluble vascular cell adhesion molecule-1 (sVCAM) levels [16,18].

Given these mechanisms, ESWT exhibits chondroprotective, analgesic, anti-inflammatory, anti-apoptotic, tissue-regenerative, and neuroregenerative properties, in addition to promoting neovascularization [16]. Scientific evidence suggests that shock waves can stimulate regenerative processes in the skin, nervous tissue, muscles, and even bones [13,14]. However, the extent of their penetration and propagation across different tissue types remains poorly understood. Further experimental research is needed to elucidate these characteristics, ensuring a safer and more effective application of shock wave therapy by healthcare professionals.

In this context, the present study aims to evaluate the penetration depth of f-ESWT in both soft and bone tissues, as well as to analyze the propagation of shock waves through biological tissues and gel pads and to evaluate its implications for safe and effective clinical application in musculoskeletal therapy. We hypothesize that f-ESWT can penetrate both soft and bone tissues but with varying degrees of energy transmission depending on tissue density and wave frequency.

## Materials and methods

Study design

This in-vitro observational study aimed to evaluate the penetration and propagation of f-ESWT in soft and bone tissues, as well as to analyze the transmission of shock waves through biological tissues and gel pads. The experiment was conducted at the Laboratory of the Institute of Orthopedics and Traumatology of the Hospital das Clínicas, Faculdade de Medicina, Universidade de São Paulo (IOT-HCFMUSP). Since this study was performed in an in-vitro setting, ethical committee approval was not required.

We conducted a total of 40 trials to evaluate the penetration of f-ESWT across different tissue types and experimental setups. Specifically, eight different frequency settings (1 Hz, 2 Hz, 3 Hz, 4 Hz, 5 Hz, 6 Hz, 7 Hz, and 8 Hz) were tested for each material - ham (presunto), human scapula, and human femur. Additionally, two applicator conditions were employed: the F10G10 applicator with a 1 cm gel pad and the F10G10 applicator with a 0 cm gel pad, both without gel. This experimental design resulted in a total of 40 trials.

The visual effects - such as the formation of bubbles and the appearance of light beams - would serve as indirect measures of the shock wave penetration depth. The visual observations were analyzed qualitatively to infer the wave’s penetration behavior into the different materials under the specified conditions. The setup included precise alignment of the shock wave device, and each trial was conducted under controlled environmental conditions to ensure consistency in the delivery of the shock waves.

Experiment description

A piezoelectric shock wave device (PW2) from Richard Wolf and ELvation® Medical, equipped with F7G3, F6G10 and F10G10 applicators, was used to assess shock wave penetration and visual propagation. Gel pads of different thicknesses (0 cm, 1 cm, 6 cm, and 9 cm) were selected to evaluate their impact on wave transmission.

To enhance the visualization of shock wave propagation, the following parameters were applied: Pulse frequency: 1 Hz to 8 Hz (maximum for the applicator); the EFD of the shock wave therapy was progressively increased across trials until the visual effects of bubble formation and light beams were observed. Starting with lower energy flux densities, the intensity was gradually elevated at each frequency setting (1 Hz to 8 Hz) until the desired visual indicators appeared, which were indicative of the shock wave propagation and penetration into the tissue samples.

The device was placed inside a 1 m³ aquarium filled with water containing suspended dirt and dust particles, allowing the detection of bubble formation or particle movement as indicators of shock wave transmission. The experiment was conducted in a dark environment with minimal lighting, focusing illumination on the water region to enhance the visibility of particle displacement.

Shock waves were tested under different conditions: water only; soft tissue simulation: 8 cm thick ham slice; bone tissue samples: human scapula and femurs (donated by the Structural Topography of Human Anatomy Department, Faculdade de Medicina, Universidade de São Paulo).

Additionally, to evaluate wave propagation through the piezoelectric generator, the experiment was performed using applicators with and without gel between the gel pad and the applicator. The movement of suspended particles in the water was observed as an indirect measure of wave transmission efficiency.

High-resolution images and videos were captured to document the mechanical wave penetration across different tissues.

## Results

We observed the formation of bubbles and light beams as visual effects indicating the propagation of shock waves, but these phenomena were only apparent at the 8 Hz frequency setting. At this frequency, the maximum EFD was recorded at 0.710 mJ/mm² with the F10G6 applicator and 0.323 mJ/mm² with the F10G10 applicator. These visual indicators were used as indirect measures of wave penetration, providing insights into the interaction between the shock waves and the tissue samples at varying energy densities. Notably, no such visual effects were observed at other frequency settings (1 Hz to 7 Hz), suggesting that the 8 Hz frequency, in combination with the highest EFD, was critical for the manifestation of these visual phenomena.

Experiment 1: Shockwave propagation without gel

In the first experiment, the propagation of focused shock waves through the piezoelectric generator was tested using applicators without gel between the applicator and the gel pad. No bubbles or visible light beams were observed during the application of the shock waves, indicating an absence of detectable propagation under these conditions (Videos 1-2).

**Video 1:** F10G10 applicator with a 1 cm gel pad without gel - no bubbles or light beams were observed during the application.

**Video 2:** F7G3 applicator with a 0 cm gel pad without gel – no bubbles or light beams were observed during the application.

Experiment 2: Shockwave propagation in soft tissue simulation

In the second experiment, an 8 cm thick ham slice was tested using the F10G10 applicator with a 9 cm gel pad, this time applying gel between the applicator and the gel pad. Bubbles and light beams were observed, confirming the propagation of focused shock waves through soft muscle-tendon tissues (Video 3).

**Video 3:** F10G10 applicator with a 9 cm gel pad with gel and 8 cm ham – bubbles and light beams were observed during the application.

Experiment 3: Shockwave propagation in bone tissue - Scapula

In the third experiment, a 3-cm thick piece of human scapula (dry bone) was tested using the F10G6 applicator with a 6-cm gel pad, with gel applied between the applicator and the gel pad. Bubbles and light beams were observed, confirming the propagation of focused shock waves through thin bone structures (Video 4).

**Video 4:** F10G6 applicator with a 6 cm gel pad with gel and a 3 cm human shoulder blade (dry bone) - bubbles and light beams were observed during the application.

Experiment 4: Shockwave propagation in bone tissue - Femur

In the fourth experiment, a 5-cm thick piece of human femur (dry bone) was tested using the F10G6 applicator with a 6-cm gel pad, with gel placed between the applicator and the gel pad. Bubbles and light beams were observed, indicating the propagation of focused shock waves through thin bone structures (Video 5).

**Video 5:** F10G6 applicator with a 6 cm gel pad with gel and 5 cm human femur (dry bone) - bubbles and light beams were observed during the application.

## Discussion

Shock waves are mechanical waves that propagate through a medium capable of deformation or density alteration. In this context, water and human tissues are considered: water serves as the standard medium for measurements, while human tissues are the primary target of ESWT in orthopedic applications [19].

When evaluating the propagation of shock waves in water, impedance is determined solely by energy levels. However, in biological tissues, additional factors come into play, as variations in tissue density further influence impedance. Impedance, in this case, is defined as the product of the medium’s density and the wave’s velocity [19,20].

Shock waves are widely used for therapeutic purposes in various tissues, including nervous tissues, skin, muscles, and bones [13,14,21]. Sağir et al. observed an increase in the number of axons in rats treated with both focused and radial shock waves, with the regenerative effect being more pronounced with the use of focused shock waves compared to radial waves [21]. Park et al. reported remyelination of the peripheral nerve and recovery of gait following the application of shock waves in a sciatic nerve injury model in rats [22]. Additional studies demonstrated that f-ESWT induced an increased proliferative profile in motor Schwann cells, while sensory Schwann cells acquired the ability to promote neurite outgrowth and express markers associated with myelination [23]. Among the various applications in the nervous system, shock wave therapy has been used in the treatment of carpal tunnel syndrome [24,25], spasticity [26], Morton’s neuroma [27,28], and some preliminary studies have investigated its potential in spinal cord injury rehabilitation [29-31].

ESWT, due to its ability to promote regeneration through the activation of stem cells, is applied in wound healing [32], the treatment of severe conditions such as Fournier’s Syndrome [33], facial rejuvenation [34], and cellulite treatment in the aesthetic field [35].

Another prominent application of shock wave therapy is in tendon and bone pathologies. This is because f-ESWT has been shown in studies by Kisch et al. to increase blood flow in the muscles of rats, suggesting that, when administered in a series, this beneficial effect is sustained [36]. A study involving elite soccer players demonstrated that shock wave therapy is a safe and effective treatment for acute muscle injuries of types 1a and 2b, in addition to preventing more severe structural muscle injuries [37]. Cho et al. elucidated the anabolic effects of shock waves in patients with sarcopenia [38]. By using radial shock waves, a significant increase in appendicular skeletal muscle mass index was observed. Additionally, the Timed Up-and-Go test and Sit-to-Stand tests revealed a notable improvement in the group treated with r-ESWT.

Studies have shown that both radial and focused shock waves exhibit anti-inflammatory, anti-fibrotic, analgesic, and regenerative effects. These therapies can stimulate local collagen production (types I and III) and soften tissue densification. These effects are attributed to the increase in fibroblasts, with modulation of TGF-β1, increased eNOS, nitric oxide (NO), and VEGF [39-42].

In bone tissue, there are several applications for the treatment of delayed bone healing, stress fractures [43], non-union [44,45], avascular necrosis without joint disruption [46], osteochondritis dissecans, and even osteoporosis [47], without joint damage. Similar to other tissues, bone tissue exhibits the phenomenon of mechanotransduction, a process by which cells convert mechanical stimuli into a chemical response. This can occur in specialized mechanosensory cells (mechanoreceptors) as well as in parenchymal cells, whose primary function is not mechanosensitive. Through biochemical signaling, gene expression and the synthesis of proteins such as integrins (α1β5, a transmembrane integrin involved in the interaction between the extracellular matrix and the cell) and osteoblasts are modulated, alongside the inhibition of miR-138, leading to increased phosphorylation and subsequent activation of FAK, mTOR activation, and subsequent S6K protein activation. This leads to an elevation in K+ and intracellular Ca2+ influx, triggering intracellular signaling cascades with FAK phosphorylation that activate ERK1/2 through MEK1/2, ultimately improving osteoblast adhesion, distribution, and migration, thereby facilitating fracture consolidation. Additionally, the activation of ERK and p38/MAPK pathways boosts the mitogenic activity dedicated to chondrogenesis and osteogenesis, while also increasing the expression of RUNX-2, a key transcription factor involved in osteogenesis [48].

The properties of shock waves and pressure waves that elicit biological effects remain incompletely understood, with multiple physical phenomena being observed. In addition to pressure gradient fluctuations, temperature variations, and cavitation effects also occur. However, the specific contributions of these factors to clinical outcomes are still under investigation, and no definitive conclusions have been reached [20].

Our experiment demonstrated that shock waves have the potential not only to reach the target tissue but also to penetrate it and affect adjacent structures, as evidenced by the visible light beam and cavitation bubbles. This is an important consideration in the application of shock wave therapy, as vital organs may be impacted during treatment.

According to the International Society for Medical Shockwave Treatment, one of the contraindications for applying high-energy shock waves is in the thoracic area, primarily due to the risk of affecting the lungs [49]. Another well-known contraindication is the application in pregnant patients, due to the potential risk of affecting the fetus through the shock waves, which could lead to undesirable effects on the pregnancy.

In our experiment, radial shock waves were not considered, as their visual effects could not be observed due to the progressive attenuation of the acoustic field during propagation. Although impulses are initially directed in a single direction, they disperse as they travel, leading to a reduction in intensity as they penetrate tissues.

In contrast, focal devices can reach depths greater than 10 cm, whereas radial devices exhibit a significant decline in energy at approximately 1.5 cm, indicating a more superficial action. However, this does not exclude the possibility that superficial applications may still induce beneficial effects in deeper tissues [19,20].

Additionally, the characteristics of shock waves vary depending on the type of generator used-whether electrohydraulic, electromagnetic, or piezoelectric. Further studies utilizing different generators may help elucidate the penetration and propagation patterns of focal shock waves across various tissue types [19,20].

Future physical experiments may assess wave propagation using hydrophones, with results displayed as time-pressure graphs, as previously conducted in other studies. Acoustic fields can be measured within defined regions, particularly at the center of focal zones, and certain parameters, such as EFD, can be derived from these measurements. Additionally, impulse could be explored as a potential parameter for further investigation as suggested by Auersperg and Trieb [19].

The experiment also presents the possibility of generating a placebo applicator for future studies utilizing the piezoelectric generator in question. This is based on the fact that the absence of gel between the gel pad and the applicator prevents the propagation of shock waves.

Despite the wide range of applications, indications, and contraindications of shock wave therapy, it is crucial for healthcare professionals to have a solid understanding of the anatomical structures to be treated, as well as their depths. Complementary diagnostic tools, such as ultrasonography, X-rays, CT scans, or MRIs, may be helpful in accurately targeting the focal point for treatment.

Our study’s results suggest that focused shock waves are capable of penetrating long bones like the femur, dense muscle layers (as observed with ham), and thinner bones, such as the scapula. However, it is important to note that the visible beam observed in our experiment only concentrates the focal point of the shock wave. There exists a much larger energy “penumbra” or focal zone that is not visible but still contains shock wave energy. This emphasizes the importance of careful and precise application by healthcare professionals to ensure safe and effective treatment.

One significant limitation of this study is the reliance on visual observations of bubble formation and light beams as indirect indicators of shock wave penetration, rather than quantitative measurements of depth or energy loss. The absence of pressure sensors or hydrophones to measure wave intensity limits the ability to compare results with existing literature and apply them precisely to clinical settings. Future research should incorporate these quantitative metrics, such as energy transmission measurements using pressure sensors, in collaboration with manufacturers, to quantify how much energy passes through different tissues and how much is reflected upon reaching specific tissues. Additionally, the use of a cadaver femur may have impacted the results, as it likely lost its bone marrow architecture, making it difficult to guarantee that in vivo results would be consistent with in-vitro findings. While the study is methodologically sound in its visual documentation and therapeutic safety considerations, these factors underscore the need for more objective and reproducible data in future investigations. Addressing these gaps by incorporating precise penetration depth measurements, energy quantification, and clinical validation would strengthen the study's impact and applicability, ensuring a more accurate and reproducible understanding of shock wave therapy.

## Conclusions

This study demonstrates that f-ESWT can effectively penetrate both soft and bone tissues, with depth of penetration varying according to tissue composition and wave intensity. The findings highlight the critical role of an appropriate coupling medium, such as gel, in ensuring effective wave propagation in piezoelectric shock wave devices. Additionally, the results reinforce the importance of anatomical knowledge for optimizing the safe and precise application of f-ESWT in clinical settings. Understanding the interaction between shock waves and different tissue types contributes to refining treatment protocols and improving therapeutic outcomes. In conclusion, while this study provides valuable insights into shock wave penetration across different tissues, future research should incorporate precise penetration depth measurements, energy quantification, and clinical validation to strengthen the study’s impact and applicability. These advancements would not only enhance the reproducibility and precision of the findings but also facilitate more accurate comparisons with existing literature and improve the clinical relevance of shock wave therapy.
